# Enhanced Antioxidant, Hyaluronidase, and Collagenase Inhibitory Activities of Glutinous Rice Husk Extract by Aqueous Enzymatic Extraction

**DOI:** 10.3390/molecules27103317

**Published:** 2022-05-21

**Authors:** Sudarat Jiamphun, Wantida Chaiyana

**Affiliations:** 1 Department of Pharmaceutical Science, Faculty of Pharmacy, Chiang Mai University, Chiang Mai 50200, Thailand; jp.plaifah@gmail.com; 2 Research Center of Pharmaceutical Nanotechnology, Chiang Mai University, Chiang Mai 50200, Thailand; 3 Innovation Center for Holistic Health, Nutraceuticals and Cosmeceuticals, Faculty of Pharmacy, Chiang Mai University, Chiang Mai 50200, Thailand

**Keywords:** *Oryza sativa*, aqueous enzymatic extraction, antioxidant, hyaluronidase, matrix metalloproteinase-1, rice husk

## Abstract

In this research, we aimed to compare the biological activities related to cosmeceutical applications of glutinous rice husk extracted by aqueous enzymatic extraction (AEE) and conventional solvent extraction. Cellulase enzymes were used to assist the extraction process. The vanillic and ferulic acid contents of each extract were investigated by high-performance liquid chromatography, and their antioxidant and anti-aging activities were investigated by spectrophotometric methods. The irritation effects of each extract were investigated by the hen’s egg test on chorioallantoic membrane. The rice husk extract from AEE using 0.5% *w*/*w* of cellulase (CE0.5) contained the significantly highest content of vanillic and ferulic acid (*p* < 0.05), which were responsible for its biological activities. CE0.5 was the most potent antioxidant via radical scavenging activities, and possessed the most potent anti-skin wrinkle effect via collagenase inhibition. Aside from the superior biological activities, the rice husk extracts from AEE were safer than those from solvent extraction, even when 95% *v*/*v* ethanol was used. Therefore, AEE is suggested as a green extraction method that can be used instead of the traditional solvent extraction technique given its higher yield and high quality of bioactive compounds. Additionally, CE0.5 is proposed as a potential source of natural antioxidants and anti-aging properties for further development of anti-wrinkle products.

## 1. Introduction

Rice is a staple food in many Asian countries, feeding more than half the world’s population due to its richness in essential vitamins, fiber, minerals, and bioactive nutrients [1,2,3]. Because rice is an important commercial crop worldwide, numerous agricultural wastes are generated throughout the rice production process in various rice-producing nations across the world [4]. There are several parts of rice disposed of as waste, e.g., husks, bran, polishing, and broken rice [1]. These wastes are low-priced and lack economic interest, especially rice husk, since it is inedible [2]. Furthermore, the removal of rice husks during rice refinement causes a disposal difficulty due to its low density, which leads to handling and transportation obstacles [4]. Moreover, rice husk contains cellulose and lignin content as high as 75–90% and, thus, it is not suitable for animal feeding [1]. Approximately 120 million tons of rice husk are globally produced each year [4]. The combustion of rice husk in the ambient environment produces a residue known as rice husk ash, as well as air pollution, which is becoming a serious health issue affecting millions of people globally [5]. Although there have been some efforts to utilize rice husk, such as reusing endeavors in the production of silica, silicate, silicon carbide, and refractory and insulating materials, the use and application of rice husk remain limited [4]. Regarding the low cost and availability, the utilization of rice husk is of interest.

There are numerous varieties of rice, including white rice and pigmented rice, e.g., black, brown, purple, and red [1,3]. *Oryza sativa* L. is the most consumed rice species worldwide [2]. Glutinous rice (*Oryza sativa* var. glutinosa), which mainly consists of amylopectin, is always sticky when cooked or steamed, and is thus called sticky rice. Glutinous rice is one of the major cultivated rice varieties with long-standing cultural importance in Asia [1]. Thailand is the largest glutinous rice exporter in the world, as it is grown in every part of the country, especially in the northern and north-eastern regions [2,3]. The annual average glutinous rice output is about 4 million tons of milled rice, or around 17.5% of overall production [3]. Apart from Asia, glutinous rice is also found on four other continents, namely, South America, North America, Africa, and Europe [3]. Therefore, it can be stated that glutinous rice is widely known, and investigations into the applications of glutinous rice husk, which would enhance its economic value, would be of interest.

For the extraction of bioactive chemicals from plant materials, a range of extraction techniques have been utilized, which may result in extracts with varying extraction yields and biological activities depending on the method used [6,7]. One of the most prevalent traditional extraction procedures is solvent extraction, which uses an organic solvent to extract bioactive chemicals from plant cells. Although solvent extraction is widely used, there are some limitations, including low extraction yields, long extraction periods, low quality of the extract due to the heat exposure during the solvent removal process, and the remaining residue of the extracting solvent. Furthermore, traditional extraction, which uses harsh chemicals for a lengthy period of time, has disadvantages in terms of cost and environmental risk [8,9]. Therefore, green extraction methods are currently being developed to overcome these limitations. Aqueous enzymatic extraction (AEE) is a green extraction technology that can reduce extraction time while improving product quality [9]. AEE has been widely used for plant oil extraction, such as coconut oil [10], soybean oil [11], sesame oil [12], and pumpkin seed oil [13]. AEE has also been employed for the extraction of hydrophilic substances, including protein and phenolic compounds, particularly from plant material with a strong structure, such as pistachio green hull [14]. Therefore, AEE is of interest to be used for the bioactive extraction of glutinous rice husk, which has tough and woody qualities. This study hence aimed to compare the safety and the potential of glutinous rice husk extracted by AEE with the conventional solvent extraction method to be used as an antioxidant, hyaluronidase, and matrix metalloproteinase-1 (MMP-1) inhibitor for further applications in cosmetic/cosmeceutical industries. The research on the biological activities of glutinous rice husk extract could be beneficial for consumers who seek to effectively benefit from natural products, especially those extracted by AEE, which is a green chemistry approach.

## 2. Results and Discussion

### 2.1. Glutinous Rice Husk Extracts

**Figure 1:**
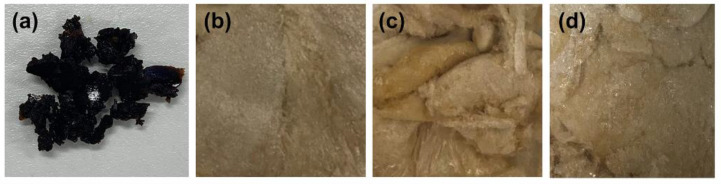
External appearance of glutinous rice husk extracts extracted by 95% *v*/*v* ethanol (EtOH) (**a**), 0.5% *w*/*w* cellulase aqueous solution (CE0.5) (**b**), 1.0% *w*/*w* cellulase aqueous solution (CE1.0) (**c**), and 1.5% *w*/*w* cellulase aqueous solution (CE1.5) (**d**).

Glutinous rice husk extracted using solvent extraction and AEE yielded different external appearances of the extracts. The ethanolic extract (EtOH) from solvent extraction was a dark brown semisolid mass, whereas all extracts from AEE using cellulase were dried powder with a light brown color, as shown in Figure 1. AEE yielded significantly higher content of glutinous rice husk extract when compared with the solvent extraction (*p* < 0.05). Although different cellulase concentrations (0.5, 1.0, and 1.5% *w*/*w*) produced extracts with the same external appearance, the higher cellulase concentration used in the AEE process yielded significantly higher content of the extracts, as shown in Table 1. This is likely due to the destruction of the plant cell walls by cellulase, leading to the higher extraction through the cell wall, and finally the enhancement of the bioactive constituents released from the plant cells [15]. Previous studies also reported that the AEE enabled an increasing yield of bioactive compounds from plants [9,16], along with a reduction in extraction time [17]. This was in agreement with the present study, which revealed that AEE using cellulase could produce a higher yield with a shorter extraction duration compared to the solvent extraction. Only 24 h was required in the AEE, whereas 9 days was required for the maceration in the solvent extraction process. Moreover, the yields of the extracts, as shown in Table 1, indicate that AEE is a replicable and generalizable approach. Regarding the low concentration of cellulase enzymes used in the extraction process, AEE is cost-effective.

Recently, there have been several types of enzymes used in AEE, e.g., cellulase, hemicellulases, arabanase, β-glucanase, pectinase, protease, and xylanase [8,13,18]. Cellulase (EC 3.2.1.4) from *Aspergillus niger* was used for the AEE of rice husk in the present study since it is one of the leading enzymes commercially produced for various industrial applications, and the enzymes from microbial sources are more stable than those from animals and plants [19,20,21]. Furthermore, cellulase has an ability to remove plant cell walls and crude fiber, resulting in the enhanced release of bioactive compounds, such as phenolic compounds, flavoring materials, essential oils, fatty acids, carotenoids, etc. [13,22,23,24]. Therefore, AEE using cellulase, which is one of the green extraction technologies, can be used as an alternative to conventional solvent extraction. Apart from a higher yield of compounds extracted and a shorter extracting duration, AEE is known as a safe technology and is also environmentally friendly [9,15,17,25].

### 2.2. Chemical Composition of Glutinous Rice Husk Extracts

**Figure 2:**
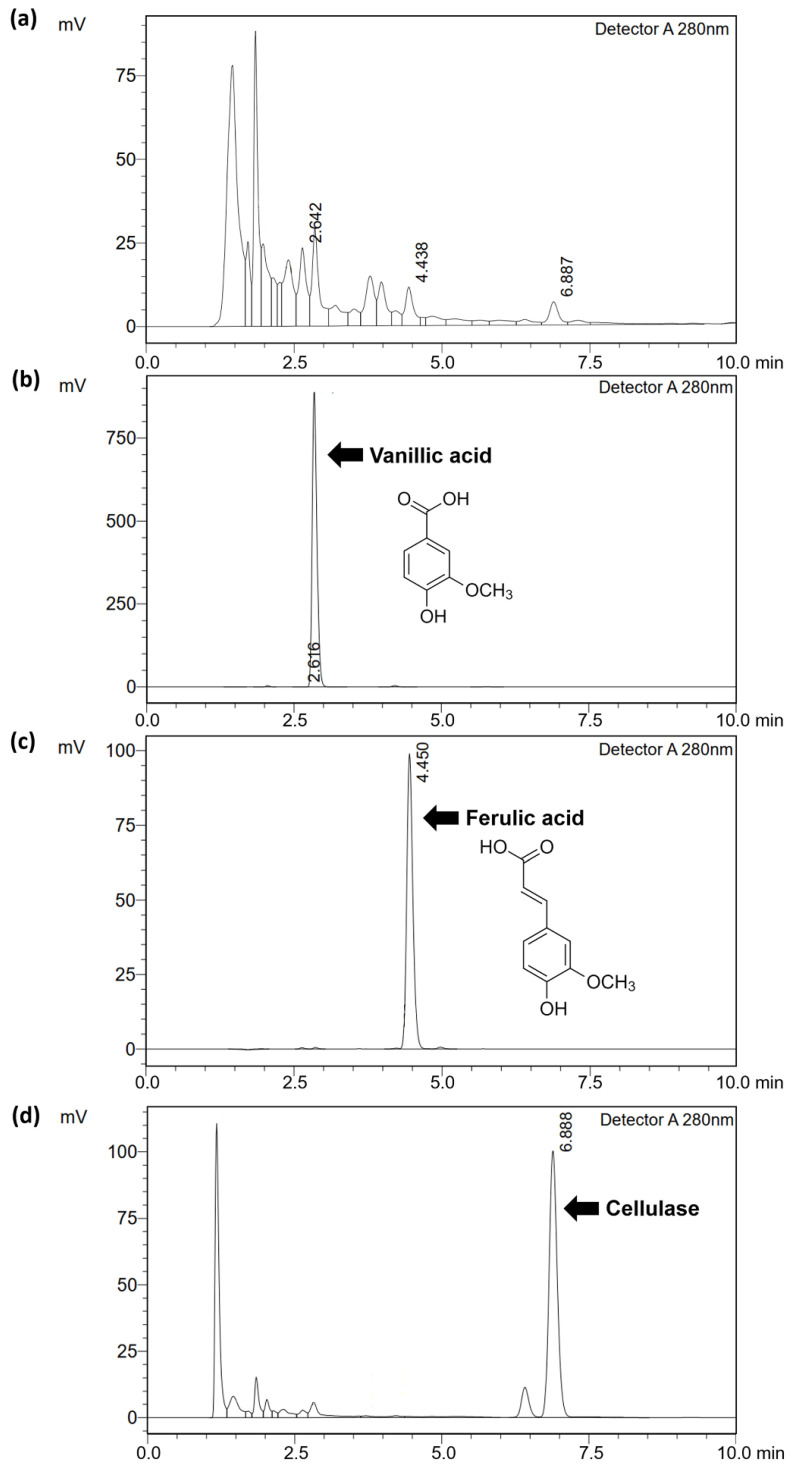
HPLC chromatograms of glutinous rice husk extracts extracted by (**a**) 0.5% *w*/*w* cellulase aqueous solution (**b**) vanillic acid (**c**) ferulic acid, and (**d**) cellulase.

The total phenolic contents of glutinous rice husk extracts, as shown in Table 2, indicate that EtOH contained the significantly highest phenolic content, followed by CE0.5, CE1.5, and CE1.0. These results are in line with previous studies, which reported that rice husk contains a variety of phenolic acids, including hydroxybenzoic acid and hydrocinnamic acid groups. Vanillic acid was the most abundant compound among different hydroxybenzoic acids, whereas ferulic acid was the most abundant compound among different hydrocinnamic acids [26]. Therefore, rice husk extracts were investigated for the content of vanillic acid and ferulic acid. Furthermore, the residues of the cellulase enzyme in the glutinous rice husk extracts from AEE were also investigated. The high-performance liquid chromatography (HPLC) chromatograms of rice husk extracts, vanillic acid, ferulic acid, and cellulase are shown in Figure 2. The rice husk extracts contained various chemical constituents, as evidenced by numerous peaks in the HPLC chromatograms. The peaks detected at around 2.6, 4.4, and 6.8 min were defined as vanillic acid, ferulic acid, and cellulase enzyme, respectively, since they were detected at the same retention time as the pure standard compounds. However, HPLC chromatogram showed peaks at retention time less than 2.5 min, indicating the presence of other compounds at higher concentrations. These unknowns could be phenolic compounds [26]. Nevertheless, further analysis was suggested in order to reveal the chemical profile of the glutinous rice husk extracts.

**Figure 3:**
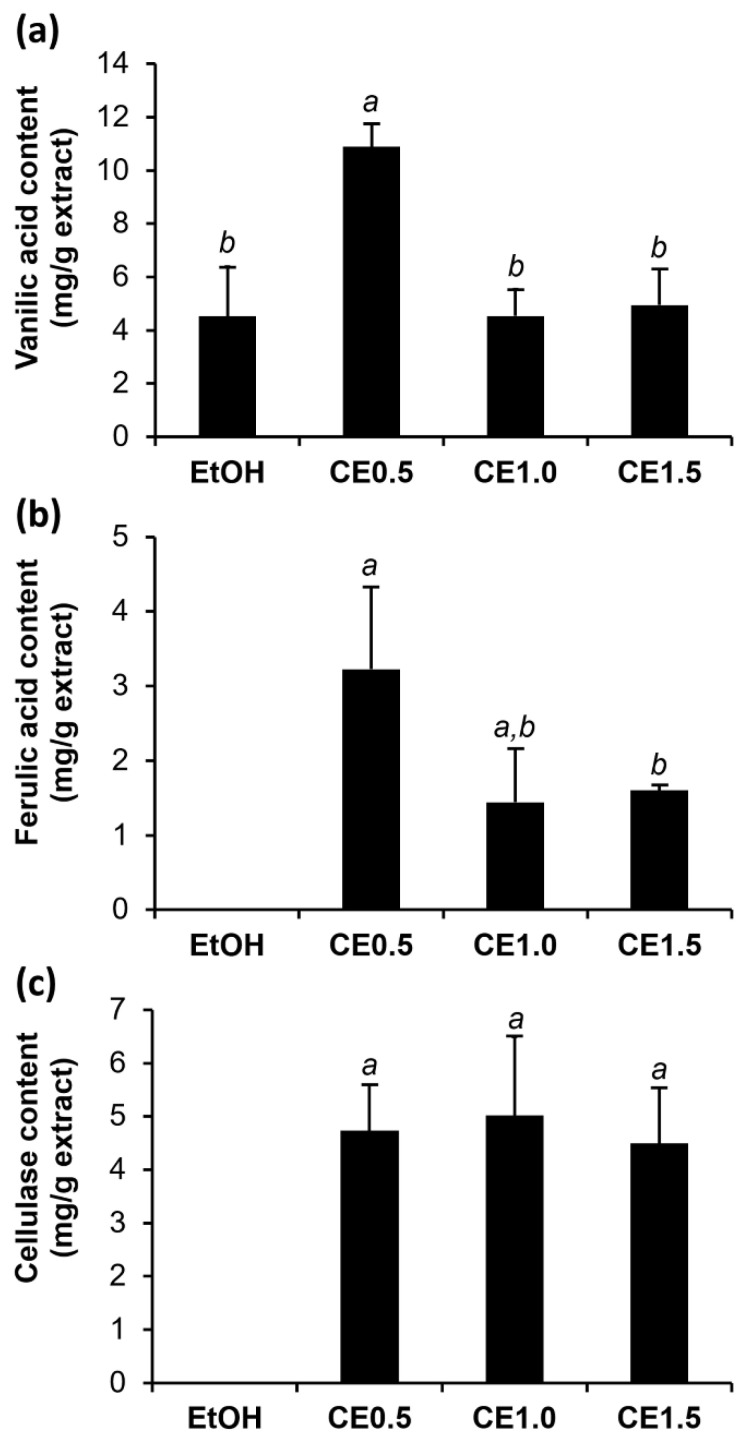
Content of vanillic acid (**a**), ferulic acid (**b**), and cellulase (**c**) of glutinous rice husk extracts extracted by 95% *v*/*v* ethanol (EtOH), 0.5% *w*/*w* cellulase aqueous solution (CE0.5), 1.0% *w*/*w* cellulase aqueous solution (CE1.0), and 1.5% *w*/*w* cellulase aqueous solution (CE1.5). Different letters, a and b, denote significant differences in the content of vanillic acid, ferulic acid, and cellulase among various glutinous rice husk extracts analyzed using one-way ANOVA with Tukey’s post hoc test (*p* < 0.05).

The contents of each compound in the rice husk extracts are shown in Figure 3. Vanillic acid was detected the most in CE0.5 (10.9 ± 0.9 mg of vanillic acid per gram of extract), which was significantly higher than that of EtOH (4.5 ± 1.9 mg of vanillic acid per gram of extract) (*p* < 0.05). In contrast, ferulic acid was detected in only the rice husk extracts from AEE, but there was no ferulic acid detected in EtOH.

The results aligned with the previous study by Butsat and Siriamornpun (2010), who found vanillic acid to be the most dominant phenolic acid in rice husk fractions, whereas ferulic acid was found as a minor constituent in the rice husk [27]. Moreover, rice husk was reported as a rich source of vanillic acid, compared to rice bran, brown rice, or milled rice [27]. Despite the fact that most phenolic compounds have limited water solubility, vanillic acid and ferulic acid were extracted using an aqueous solution with the assistance of the cellulase enzyme. The previous study reported that the solubility of vanillic acid as 100 x1^exp^ in mole fraction when x1 was 6.15 in ethanol but only 4.66 × 10^−2^ in water at a temperature of 323.15 K (50 °C) [28]. Similarly, the solubility of ferulic acid in ethanol was 3.08 × 10^−2^ in mole fraction but only 1.36 × 10^−4^ in water at the temperature of 318.2 K (~45 °C) [29]. However, the present study noted that AEE enhanced the extraction efficiency of both vanillic acid and ferulic acid compared to the solvent extraction using ethanol. The likely explanation is the cellulolytic effect of cellulase to degrade cellulose, a major polysaccharide constituent of plant cell walls, which results in the higher extraction efficiency and increased extraction yield of the extracts of plant origin [30]. Furthermore, the results were in accordance with the previous report which revealed that the phenolic contents of cellulase-treated rice husk were enhanced compared with untreated [26]. In addition, the slight acidic pH (6.5) of the cellulase enzyme aqueous solution used in the AEE process might lead the phenolic compounds to be in the ionized form, which is more soluble in the aqueous solution used as an extracting solvent. Therefore, AEE is suggested as a safe, environmentally friendly, and economical alternative extraction method for the production of rice husk extract [31,32].

Among the rice husk extracts, it is interesting that CE0.5 had the highest concentrations of vanillic acid (10.9 ± 0.9 mg of vanillic acid per gram of extract) and ferulic acid (3.2 ± 1.1 mg of ferulic acid per gram of extract) (*p* < 0.05). Therefore, a cellulase dose higher than 0.5% *w*/*w* was unnecessary for the extraction of phenolic compounds. Normally, higher enzyme concentrations lead to a higher extraction rate, but a higher concentration of the enzymes than the optimal concentration would be worthless due to the maximum interactions between enzymes and their substrate molecules [33]. Furthermore, the optimal enzyme concentration had to be investigated to attain the optimal balance between the extraction efficiency and the cost [34]. Several studies observed that the medium enzyme concentration favored extraction [34,35,36]. In brief, the present study suggested a 0.5% *w*/*w* concentration of cellulase enzyme for the AEE process.

### 2.3. Antioxidant Activities of Glutinous Rice Husk Extracts

Antioxidant activities of glutinous rice husk extracts were investigated by means of radical scavenging activity on 2,2-diphenyl-1-picrylhydrazyl radical (DPPH^•^) and 2,2-azino-bis3-ethylbenzothiazoline-6-sulfonic acid radical (ABTS^•+^) and reported as the concentration of glutinous rice husk extracts inhibiting the activity of DPPH^•^ by 50% (IC_50_) and Trolox equivalent antioxidant capacity (TEAC), respectively. Furthermore, the ferric reducing antioxidant power (FRAP), i.e., the reducing ability through the reduction of ferric iron (Fe^3+^) to ferrous iron (Fe^2+^), of the glutinous rice husk extracts was investigated and reported as equivalent concentration (EC_1_). Regarding the antioxidant activities of glutinous rice husk extracts, as presented in Table 3, it was noted that the glutinous rice husk extracts exhibited potent radical scavenging activities, but low ferric reducing antioxidant power. Among different glutinous rice husk extracts, the extracts from AEE exhibited both DPPH^•^ and ABTS^•+^ radical scavenging activities, whereas EtOH scavenged only the activity of the DPPH^•^ radical, but not the ABTS^•+^ radical. The cellulase enzyme had no effect on the antioxidant activity in all the tests. Ferulic acid was proposed as the component responsible for the radical scavenging activities and reducing power of glutinous rice husk extracts due to its potent activity. These results correspond with previous studies, which reported that enzyme-aided extraction significantly increased the antioxidant activity of the extract since it promoted the release of antioxidant polyphenols [26,36,37,38]. In the present study, CE0.5 was identified as the most potent extract in terms of radical scavenging activities against DPPH^•^ and ABTS^•+^ radical. Its radical scavenging activities were equivalent to ascorbic acid, a well-known and extensively employed natural antioxidant. Therefore, CE0.5 could be used as natural extract from green extraction technology as an active ingredient or additive in a variety of areas, including pharmaceutical, cosmetic, cosmeceutical, and food industries.

### 2.4. Anti-Aging Activities of Glutinous Rice Husk Extracts

**Figure 4:**
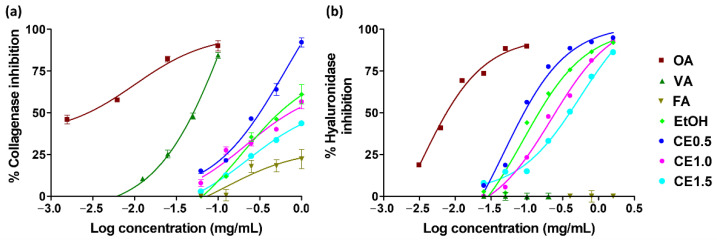
Dose–response curve of the inhibition of collagenase (**a**) and hyaluronidase (**b**) of oleanolic acid (OA), vanillic acid (VA), ferulic acid (FA), and glutinous rice husk extracts extracted by 95% *v*/*v* ethanol (EtOH), 0.5% *w*/*w* cellulase aqueous solution (CE0.5), 1.0% *w*/*w* cellulase aqueous solution (CE1.0), and 1.5% *w*/*w* cellulase aqueous solution (CE1.5).

The anti-aging activities of glutinous rice husk extracts were investigated by means of collagenase and hyaluronidase inhibition, which are shown in Figure 4. Among different glutinous rice husk extracts, CE0.5 was the significantly most potent inhibitor on collagenase and hyaluronidase activities, with IC_50_ values of 0.4 ± 0.03 mg/mL and 47.5 ± 0.7 µg/mL, respectively (*p* < 0.05). Vanillic acid was found as the major component responsible for anti-collagenase activity of the extract since it possessed a potent inhibition with the IC_50_ value of 57.6 ± 1.4 µg/mL. In contrast, EtOH, which contained the significantly highest content of total phenolic compounds, did not show the strongest biological activities. Therefore, the content of phenolic compounds was not related to their biological activities, and it could be highlighted that although there were various phenolic compounds, vanillic was suggested as the major component that was responsible for the biological activities of glutinous rice husk extracts.

Since both collagenase and hyaluronidase are the enzymes capable of degrading collagen and hyaluronan, which are the extracellular matrices with critical roles in skin tightening and skin resilience, the inhibition of these enzymes would lead to the retardation of skin aging [39,40]. Collagen fibers have been known as the major fibrillar component of the skin dermis, a connective tissue layer that lies between the epidermis and the subcutaneous tissue [39]. Therefore, the breakdown of collagen fibers contributes to the appearance of skin wrinkles and aging [41], whereas loss of skin hydration leads to wrinkles and deeper furrows [42]. Hyaluronan, a natural moisturizing factor (NMF), is responsible for water homeostasis in human skin [43]. The reduction in NMF, the human skin’s barrier, is associated with changes in corneocyte surface [44]. CE0.5, which inhibits both collagenase and hyaluronidase, is suggested as an active ingredient in cosmetics and cosmeceuticals for the retardation of skin wrinkles.

### 2.5. In Vitro Irritation Properties of Glutinous Rice Husk Extracts

The irritation of glutinous rice husk extracts was one of the concerns that needed to be tested before using the extract in further applications for cosmetic or cosmeceutical purposes. The hen’s egg test on chorioallantoic membrane (HET-CAM) assay was used to investigate the irritation properties of glutinous rice husk extracts because it has been widely used to screen for and assess both irritation and anti-irritant properties of tested compounds [45]. In light of the public and scientific debate over animal experiments, the Draize rabbit eye test is being abandoned, and efforts have been undertaken in the international cosmetics industry over several decades to verify adequate and appropriate in vitro testing [45]. Some key results from the HET-CAM assay were compared to those obtained in direct human skin tests using the same test irritant, leading to the conclusion that an essentially comparable pattern of effectiveness was discovered in these comparative skin tests [46]. Therefore, the HET-CAM assay has been proposed as a pre-screening method for irritation tests before clinical study.

**Figure 5:**
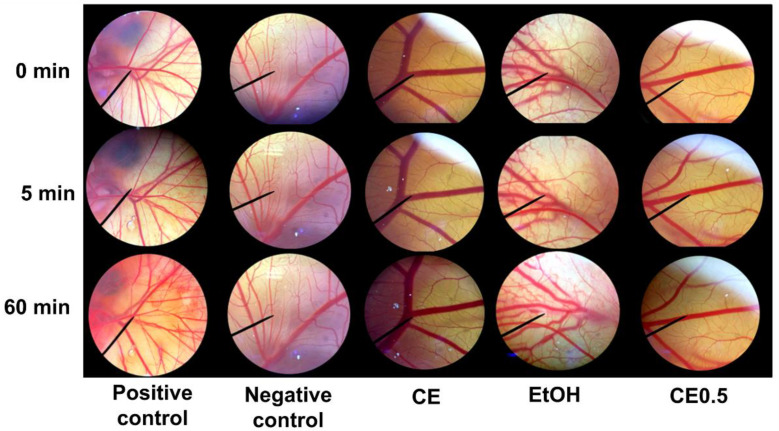
Effect of 1% *w*/*v* sodium lauryl sulfate aqueous solution (positive control), 0.9% *w*/*v* normal saline solution (negative control), cellulase enzyme (CE), and glutinous rice husk extracts extracted by 95% *v*/*v* ethanol (EtOH) and 0.5% *w*/*w* cellulase aqueous solution (CE0.5) on the hen’s egg chorioallantoic membrane before (0 min) and after exposure for 5 and 60 min.

The results shown in Figure 5 indicate that the positive control (aqueous solution of 1% *w*/*v* sodium lauryl sulfate (SLS)) induced severe irritation, with an irritation index score (IS) of 15.6 ± 0.1. Signs of irritation were triggered after the application of the positive control, notably hemorrhage, which immediately occurred once after the application, and was followed by coagulation and vascular lysis. Conversely, the negative control (normal saline solution), glutinous rice husk extracts (EtOH and CE0.5), and cellulase induced no sign of irritation on the chorioallantoic membrane (CAM) after 5 min of the application. Therefore, the IS was 0.0, referred to as “no irritation”. However, after 60 min of the applications, EtOH induced vascular lysis. As a result, it was highlighted that the glutinous rice husk extract (CE0.5) was safer and more suitable for topical application than the ethanolic extract (EtOH).

## 3. Materials and Methods

### 3.1. Plant Materials

The husk from *Oryza sativa* L. var. Niaw San-pah-tawng, which is glutinous rice, was removed from the grain during the rice production process by Chiang Mai Phon Suriya Rice Mill, Co., Ltd., Chiang Mai, Thailand, in January 2019. The glutinous rice husk, which was being discarded as waste, was obtained as a gift from Chiang Mai Phon Suriya Rice Mill, Co., Ltd., Chiang Mai, Thailand. The fresh plant materials from the rice field of Chiang Mai Phon Suriya Rice Mill, Co., Ltd. were identified and authenticated by Ms. Wannaree Charoensup, a botanist at the Herbarium, Department of Pharmaceutical Science, Faculty of Pharmacy, Chiang Mai University. The voucher specimen number 0023305 was kept at the herbarium of Faculty of Pharmacy, Chiang Mai University, Thailand. The rice husk materials were ground into fine powder and kept in sealed plastic bags until further used.

### 3.2. Chemical Materials

Cellulases (1,4-(1,3;1,4)-/3-D-glucan 4-glucanohydrolase, E.C.3.2.1.4, ≥0.3 units/mg solid, optimal pH = 6.5, optimal temperature = 50–55 °C) from *Aspergillus niger*, bovine testicular hyaluronidase (E.C.3.2.1.3.5), MMP-1 from *Clostridium histolyticum* (ChC-E.C. 3.4.23.3), ascorbic acid (vitamin C), vanillic acid, ferulic acid, 2,4,6 tripyridyl-s-triazine (TPTZ), aluminum chloride (AlCl_3_), 2,2-azino-bis3-ethylbenzothiazoline-6-sulfonic acid (ABTS), calcium chloride (CaCl_2_), ferric chloride (FeCl_3_), ferrous chloride (FeCl_2_), ferrous sulfate (FeSO_4_), Folin–Ciocalteu reagent, 6-hydroxy-2,5,7,8-tetramethylchroman-2-carboxylic acid (Trolox),quercetin, sodium lauryl sulfate (SLS), hyaluronic acid, hydrochloric acid (HCl), potassium acetate (CH_3_COOK), potassium persulphate (K_2_S_2_O_8_), sodium acetate (C_2_H_3_NaO_2_), sodium carbonate (Na_2_CO_3_), sodium chloride (NaCl), sodium dihydrogen phosphate (NaH_2_PO_4_), disodium phosphate (Na_2_HPO_4_), and sodium phosphate (Na_3_PO_4_) were purchased from Sigma-Aldrich (St. Louis, MO, USA). Bovine serum albumin (BSA) (catalog number: B14) was bought from Gibco™ (Thermo Fisher Scientific, Waltham, MA, USA). Acetic acid and ethanol were of analytical grade and purchased from Labscan, Ltd. (Dublin, Ireland). Acetonitrile was HPLC grade and purchased from Labscan, Ltd. (Dublin, Ireland). Distilled (DI) water was purchased from RCI Labscan Co., Ltd. (Bangkok, Thailand).

### 3.3. Extraction of Glutinous Rice Husk

#### 3.3.1. Solvent Extraction

Ethanol was used as an organic solvent in the solvent extraction process of glutinous rice husk due to its safety for dermal applications. In brief, 150 g of the glutinous rice husk powder was extracted by maceration in 95% *v*/*v* ethanol for 3 days, in 3 cycles at room temperature. The ratio of glutinous rice husk and the solvent was 1:5 by weight. Therefore, the amount of solvent used in each cycle was 750 g, for a total of 2250 g for 3 cycles. The resulting extract was filtered through Whatman No. 1 filter paper, and the solvents were then removed by evaporation using a rotary evaporator (Buchi Labortechnik GmbH, Essen, Germany). Crude extract of 95% *v*/*v* ethanol (EtOH) was obtained and stored in well-closed container at 4 °C until the next experiments. The extraction was repeated 3 times to ensure the consistency of the results.

#### 3.3.2. Aqueous Enzymatic Extraction

Cellulase (E.C.3.2.1.4) from *A. niger* was used in the aqueous enzymatic extraction process of glutinous rice husk. In brief, 150 g of the glutinous rice husk powder was extracted by AEE using various concentrations of cellulase aqueous solutions, including 0.5, 1.0, and 1.5% *w*/*w*. The ratio of glutinous rice husk and the solvent was 1:5 by weight. Therefore, the amount of each concentration of cellulase aqueous solutions was 750 g. The AEE was performed following the conditions of Wanyo et al. (2014) [26]. The optimal conditions for AEE were pH of 6.5, temperature of 50 °C, and duration of 24 h. Thereafter, the resulting extracts were filtered through Whatman No. 1 filter paper and dried in a freeze-dryer (FreeZone 4.5 model 7750031, Labconco, Kansas, MO, USA). Three glutinous rice husk extracts from AEE, including CE0.5, CE1.0, and CE1.5, were obtained. All extracts were stored in a well-closed container at 4 °C until the next experiments. The extraction was repeated 3 times to ensure that the results were consistent.

### 3.4. Determination of Total Phenolic Content by Folin–Ciocalteu Method

The total phenolic content of each glutinous rice husk extracts was measured using the Folin–Ciocalteu’ method according to Poomanee’s technique [47]. Gallic acid was utilized as a reference standard compound, and the results are expressed as milligrams of gallic acid equivalent per gram of each extract. In brief, the glutinous rice husk extracts was dissolved in a combination of ethanol and DI water in a volume ratio of 2:3. Then, the resulting solution of glutinous rice husk extracts was mixed with a 10-fold diluted Folin–Ciocalteu solution, followed by the addition of sodium carbonate solution. The mixture was kept at room temperature for 30 min and the absorbance was measured at 765 nm by a multimode detector (SPECTROstar Nano, BMG Labtech, Offenburg, Germany). The experiments were carried out 3 times.

### 3.5. Determination of Vanillic Acid, Ferulic Acid, and Cellulase Content by High-Performance Liquid Chromatography (HPLC)

The HPLC analysis with a Supelcosil LC-18 column (250 cm 4.6 mm, 5 m; Supelco Analytical, Bellefonte, PA, USA) and fixed temperature at 38 °C as a stationary phase was used to determine the content of vanillic acid, ferulic acid, and cellulase in glutinous rice husk extracts. The mixture of acetonitrile and 0.1% *v*/*v* acetic acid aqueous solution in a 20:80 ratio, which was previously filtered through a 0.45 m nylon filter membrane and degassed in a sonicator for 30 min, was used to elute the glutinous rice husk extracts at a flow rate of 1 mL/min. A UV detector set at 280 nm was used to detect the compounds eluted from the analysis. Subsequently, the contents of vanillic acid, ferulic acid, and cellulase were calculated using the equation constructed from the standard curves of each compound as follows: vanillic acid content (mg/g extract) = (A + 2757)/35,468B (R² = 1.000), where A is the area under the curve (AUC) of the vanillic acid peak detected around 2.8 min and B is the concentration of vanillic acid solution; ferulic acid content (mg/g extract) = (A + 4553)/22,362B (R² = 0.999), where A is the AUC of the ferulic acid peak detected around 4.5 min and B is the concentration of ferulic acid solution; cellulase content (mg/g extract) = (A + 17,205)/19,855B (R² = 0.999), where A is the AUC of the cellulase peak detected around 6.8 min and B is the concentration of cellulase solution.

### 3.6. Antioxidant Activity Determination

#### 3.6.1. 2,2-Diphenyl-1-Picrylhydrazyl (DPPH) Radical Scavenging Assay

The glutinous rice husk extracts and the reference standards were investigated for their radical scavenging activity via the DPPH assay, which was modified following the methods of Brem et al. (2004) and Chaiyana et al. (2017) [48,49]. In brief, the combination of DPPH^•^ solution and the sample solution was prepared in a 1:9 ratio into a final volume of 200 μL. After incubation in the dark for 30 min, the absorbance was measured at 520 nm by a multimode detector (SPECTROstar Nano, BMG Labtech, Offenburg, Germany). Ascorbic acid was used as a positive control. The DPPH^•^ scavenging activity was calculated using the following equation:

DPPH^•^ scavenging activity (%) = [(A − B)/A] × 100, (1)

where A is the difference between the UV absorbance of the DPPH^•^ solution and its solvents and B is the difference between the UV absorbance of the sample solution with and without the DPPH^•^ solution. IC_50_ values were calculated from the dose–response curve plotted from the DPPH^•^ scavenging activity (%) versus its log concentrations using the GraphPad/Prism program version 2.01 (GraphPad Software Inc., La Jolla, CA, USA). The positive control was ascorbic acid. The experiments were carried out 3 times.

#### 3.6.2. 2,2′-Azino-Bis(3-Ethylbenzothiazoline-6-Sulfonic Acid) (ABTS) Assay

The glutinous rice husk extracts and the reference standards were investigated for their radical scavenging activity via the ABTS assay, which was modified following the methods of Pellegrini et al. (2003) and Chaiyana et al. (2017) [49,50]. In brief, the ABTS free radical (ABTS^•+^) solution was prepared from the combination of 7 mM of ABTS solution and 2.45 mM of potassium persulphate (K_2_S_2_O_8_) solution in a 2:3 ratio and incubated at room temperature in the dark for 16 h. Thereafter, the combination of ABTS^•+^ solution and the sample solution was prepared in a 1:9 ratio into a final volume of 200 μL. After incubation for 5 min, the absorbance was measured at 750 nm by a multimode detector (SPECTROstar Nano, BMG Labtech, Offenburg, Germany). Ascorbic acid was used as a positive control. Trolox equivalent antioxidant capacity (TEAC), which is the amount of Trolox that is equivalent to 1 mg of the glutinous rice husk extracts, was calculated using the equation constructed from the standard curves of Trolox plotted with ABTS^•+^ scavenging activity versus various concentrations of Trolox. The experiments were carried out 3 times.

#### 3.6.3. Ferric Reducing Antioxidant Power (FRAP) Assay

The glutinous rice husk extracts and the reference standards were investigated for their ferric reducing antioxidant power via the FRAP assay, which was modified following the methods of Saeio et al. (2011) and Chaiyana et al. (2017) [49,51]. In brief, the FRAP reagent was prepared from the combination of 0.3 M acetate buffer pH 3.6, 20 mM FeCl_3_ solution, and 10 mM TPTZ solution in 40 mM HCl in a 10:1:1 ratio and freshly used. Thereafter, the combination of FRAP reagent and the sample solution was prepared in a 1:9 ratio into a final volume of 200 μL. After incubation for 5 min, the absorbance was measured at 595 nm by a multimode detector (SPECTROstar Nano, BMG Labtech, Offenburg, Germany). Ascorbic acid was used as a positive control. EC_1_, the amount of FeSO_4_ that is equivalent to 1 mg of the glutinous rice husk extracts, was calculated using the equation constructed from the standard curves of FeSO_4_ plotted with the UV absorbance versus various concentrations of FeSO_4_. The experiments were carried out 3 times.

### 3.7. Anti-Aging Activity Determination

#### 3.7.1. Determination of Collagenase Inhibitory Activity

The glutinous rice husk extracts and the reference standards were investigated for their collagenase inhibitory activity via the enzyme substrate assay, which was modified following the methods of Thring et al. (2009) [52]. In this experiment, 50 mM Tricine buffer pH 7.5 containing 400 mM NaCl and 10 mM CaCl_2_ was used as a solvent for the enzymatic reaction system. In brief, the combination of 0.5 units/mL collagenase and the sample solution was prepared and incubated for 15 min. Thereafter, 2.0 M FALGPA was added and immediately measured for an absorbance of 340 nm in a kinetic mode for 20 min using a multimode detector (SPECTROstar Nano, BMG Labtech, Offenburg, Germany). The collagenase inhibitory activity was calculated using the following equation:

Collagenase inhibition (%) = [(A − B)/A] × 100,(2)

where A is the reaction rate of the system without sample and B is the reaction rate of the system with sample. IC_50_ values were calculated from the dose–response curve plotted from the collagenase inhibition (%) versus its log concentrations using the GraphPad/Prism program version 2.01 (GraphPad Software Inc., La Jolla, CA, USA). The positive control was oleanolic acid. The experiments were carried out 3 times.

#### 3.7.2. Determination of Hyaluronidase Inhibitory Activity

The glutinous rice husk extracts and the reference standards were investigated for their hyaluronidase inhibitory activity via the enzyme substrate assay, which was modified following the methods of Chaiyana et al. (2019) [53]. In this experiment, phosphate buffer pH 5.3 was used as a solvent for the enzymatic reaction system. In brief, the combination of 1.5 unit/mL hyaluronidase and the sample solution was prepared and incubated at 37 °C for 10 min. Thereafter, 0.03% *w*/*v* hyaluronic acid was added and incubated again at 37 °C for 45 min. Subsequently, acid bovine serum albumin solution, composed of acetic acid, sodium acetate, and bovine serum albumin, was added. After incubation for 10 min, the mixture was measured for absorbance at 600 nm using a multimode detector (SPECTROstar Nano, BMG Labtech, Offenburg, Germany). The hyaluronidase inhibitory activity was calculated using the following equation:

Hyaluronidase inhibition (%) = [(A − B)/A] × 100,(3)

where A is the UV absorbance of the system without sample and B is the UV absorbance of the system with sample. IC_50_ values were calculated from the dose–response curve plotted from the hyaluronidase inhibition (%) versus its log concentrations using the GraphPad/Prism program version 2.01 (GraphPad Software Inc., La Jolla, CA, USA). The positive control was oleanolic acid. The experiments were carried out 3 times.

### 3.8. Irritation Test of Glutinous Rice Husk Extracts by Hen’s Egg Test Chorioallantoic Membrane (HET-CAM) Assay

The glutinous rice husk extracts and the reference standards were investigated for their irritation capacity via HET-CAM assay, which was modified following the methods of Somwongin et al. (2018) [54]. Different adverse occurrences, including hemorrhage, vascular lysis, and coagulation, on the CAM of the hen’s egg after the application of sample solution was detected and calculated for the irritation index score (IS) using the following equation:

IS = [(301 − t(h))/300 × 5] + [(301 − t(l))/300 × 7] + [(301 − t(c))/300 × 9],(4)

when the initial vascular hemorrhage occurred at time t(h), the initial vascular lysis occurred at time t(l), and the initial coagulation occurred at time t(c) [45,54]. IS was then classified as follows: 0.0–0.9 indicated no irritation, 1.0–4.9 indicated mild irritation, 5.0–8.9 indicated moderate irritation, and 9.0–21.0 indicated severe irritation. NSS (0.9% *w*/*v* NaCl) was used as a negative control and was used for dissolving all the tested compounds. On the other hand, 1% *w*/*v* SLS aqueous solution was used as a positive control. The experiments were carried out 3 times.

### 3.9. Statistical Analysis

Data are reported as mean and standard deviation (S.D.). Statistical significance was assessed using one-way analysis of variance (ANOVA) using GraphPad Prism (version 8.0, GraphPad Software). The level of significant difference was set as *p* < 0.05.

## 4. Conclusions

The findings from this research highlight AEE as a green extraction technology that can generate glutinous rice husk extracts with enhanced biological activities and is safe for skin applications. A shorter duration of the AEE was required when compared with the solvent extraction. Moreover, higher contents of bioactive components were more effectively extracted by AEE. CE0.5, which possessed the significantly highest antioxidant, anti-collagenase, and anti-hyaluronidase activities (*p* < 0.05), were safe and induced no sign of irritation on the CAM. Vanillic acid was a major compound, responsible for the collagenase inhibitory activity, whereas ferulic acid was a minor compound, responsible for the strong antioxidant activities. Given its chemical component, biological activities, and non-irritation, CE0.5 is suggested to be used as a natural anti-aging ingredient in the development of cosmetic/cosmeceutical products. This would increase the value of rice husk, which is the most common agricultural waste with hardly any commercial use. However, additional biological tests related to skin health improvement are needed, such as anti-inflammatory activity for soothing effects, anti-tyrosinase activity for whitening effects, etc. Moreover, performance tests on human volunteers are suggested.

## Figures and Tables

**Table 1:** Yields of glutinous rice husk extract.

Glutinous Rice Husk Extract	Weigh of Yield (g) ^5^	Yield (% *w*/*w*)
EtOH ^1^	2.0 ± 0.2 ^d^	1.3 ± 0.1 ^d^
CE0.5 ^2^	2.9 ± 0.1 ^c^	1.9 ± 0.1 ^c^
CE1.0 ^3^	3.6 ± 0.1 ^b^	2.4 ± 0.0 ^b^
CE1.5 ^4^	4.0 ± 0.1 ^a^	2.7 ± 0.0 ^a^

^1^ EtOH is a glutinous rice husk ethanolic extract, ^2^ CE0.5 is a glutinous rice husk extracted by 0.5% *w*/*w* cellulase aqueous solution, ^3^ CE1.0 is a glutinous rice husk extracted by 1.0% *w*/*w* cellulase aqueous solution, and ^4^ CE1.5 is a glutinous rice husk extracted by 1.5% *w*/*w* cellulase aqueous solution; ^5^ the weight of yield was from 150 g of a dried powder of glutinous rice husk material. Different letters, ^a^, ^b^, ^c^, and ^d^, denote significant differences in weight of yield or the percentage of yield among various glutinous rice husk extracts analyzed using one-way ANOVA with Tukey’s post hoc test (*p* < 0.05).

**Table 2:** The total phenolic contents of rice husk extracts.

Glutinous Rice Husk Extract	TPC (mg GAE/g Extract) ^5^
EtOH ^1^	255.8 ± 2.0 ^a^
CE0.5 ^2^	180.0 ± 1.7 ^b^
CE1.0 ^3^	92.0 ± 0.2 ^d^
CE1.5 ^4^	103.0 ± 0.8 ^c^

^1^ EtOH is a glutinous rice husk ethanolic extract, ^2^ CE0.5 is a glutinous rice husk extracted by 0.5% *w*/*w* cellulase aqueous solution, ^3^ CE1.0 is a glutinous rice husk extracted by 1.0% *w*/*w* cellulase aqueous solution, ^4^ CE1.5 is a glutinous rice husk extracted by 1.5% *w*/*w* cellulase aqueous solution, and ^5^ TPC is total phenolic content represented in the term of mg of gallic acid equivalent to 1 g of the extract. Different letters, ^a^, ^b^, ^c^, and ^d^, denote significant differences in the total phenolic content among various glutinous rice husk extracts analyzed using one-way ANOVA with Tukey’s post hoc test (*p* < 0.05).

**Table 3:** Antioxidant activities of glutinous rice husk extracts.

Samples	IC_50_ on DPPH ^1^ (µg/mL)	TEAC ^2^ (µg Trolox/mg Extract)	EC_1_ ^3^ (mM FeSO_4_/mg Extract)
AS ^4^	4.3 ± 0.2 ^a^	12.3 ± 0.0 ^a^	238.3 ± 0.2 ^a^
VA ^5^	1750.7 ± 321.0 ^c^	5.4 ± 0.1 ^c^	126.7 ± 1.3 ^c^
FA ^6^	37.9 ± 0.8 ^a^	12.3 ± 0.0 ^a^	234.5 ± 1.0 ^b^
CE ^7^	N.D. ^12^	0.0 ± 0.1 ^e^	0.0 ± 0.3 ^g^
EtOH ^8^	184.0 ± 20.8 ^a^	0.0 ± 0.8 ^e^	74.9 ± 0.4 ^d^
CE0.5 ^9^	201.3 ± 13.7 ^a^	6.5 ± 0.3 ^b^	38.1 ± 0.9 ^e^
CE1.0 ^10^	614.8 ± 17.3 ^b^	3.2 ± 0.2 ^d^	34.7 ± 1.1 ^e^
CE1.5 ^11^	847.6 ± 90.5 ^b^	3.1 ± 0.2 ^d^	21.6 ± 2.7 ^f^

^1^ IC_50_: the concentration of glutinous rice husk extracts inhibiting the activity of DPPH^•^ by 50%; ^2^ TEAC: Trolox equivalent antioxidant capacity; ^3^ EC_1_ is equivalent concentration; ^4^ AS: ascorbic acid; ^5^ VA: vanillic acid; ^6^ FA: ferulic acid; ^7^ CE: cellulase enzyme; ^8^ EtOH: ethanolic extract; ^9^ CE0.5: rice husk extracts 0.5% *w*/*w* cellulase aqueous solution; ^10^ CE1.0: rice husk extracts 1.0% *w*/*w* cellulase aqueous solution; ^11^ CE1.5: rice husk extracts 1.5% *w*/*w* cellulase aqueous solution; ^12^ N.D.: not determined. The letters ^a^, ^b^, ^c^, ^d^, ^e^, ^f^, and ^g^ denote significant differences in the antioxidant activities among samples analyzed using one-way ANOVA with Tukey’s post hoc test (*p* < 0.05).

## Data Availability

Not applicable.

## Sample Availability

Samples of the compounds are available from the authors.
